# A Geological Context in Radiation Risk Assessment to the Public

**DOI:** 10.3390/ijerph191811750

**Published:** 2022-09-17

**Authors:** Filip Jędrzejek, Katarzyna Szarłowicz, Marcin Stobiński

**Affiliations:** AGH University of Science and Technology, Faculty of Energy and Fuels, al. Mickiewicza 30, 30-059 Kraków, Poland

**Keywords:** radionuclide (NORM), geology, absorbed dose rate

## Abstract

The work aimed to show the applicability of geological studies to the investigation of radiation risk assessment due to the presence of naturally occurring radionuclides of terrestrial origin in the soil. Soil samples were taken from a Tatra Mountains area for which geological maps were available. The concentration of selected radionuclides incl. ^40^K, ^238^U and ^232^Th was determined by gamma-ray spectrometry with a HPGe-detector. Radioactivities and calculated absorbed dose rates were co-related to complex bedrock matrices based on an original methodology. The correlations were proved by performing a Principal Component Analysis (PCA). The rocks that had a significant impact on the rate of absorbed dose from the soil were strongly related to the radioactivity of the uranium series. The share of the following fractions was the most significant: granite with pegmatite, gneiss, granitoid and gneiss, coquina, marl and glauconite, hard limestone, dolomite and limestone. The rock types additionally showed good correlation with radioisotopes from the thorium series. Granitoids with potassium feldspar, on the other hand, contributed the largest share of ^40^K radioisotope content.

## 1. Introduction

Naturally occurring radionuclides of terrestrial origin are present in almost all environmental media. There are many different radioisotopes, but in practical terms only a few have sufficient abundance and radiological importance [1,2]. Most research on naturally occurring radionuclides in soils has focused on primordial ^40^K, ^238^U, ^232^Th and ^226^Ra from uranium series [3,4,5]. Radiological protection of the population and assessment of the radiological condition of the environment remain the main focuses of these studies. Possible routes of population exposure to terrestrial radionuclides contained in soils are as follows: direct exposure to external radiation, internal radiation from inhalation of airborne radionuclides and internal dose from ingestion (food and water contamination) [6,7]. This study focuses exclusively on radionuclides from the former source, where the radiation dose comes from gamma and beta radiation connected with the presence of radionuclides in the surface soil layer.

The most prominent organization that provides regular evaluation of worldwide dose rate data and periodically issues subject reports is the United Nations Scientific Committee on the Effects of Atomic Radiation (UNSCEAR). In its reports, the organization outlines methodologies for performing dose assessments and provides a reliable model for other institutions and agencies that conduct such studies in local areas. However, a key issue for a proper assessment of radiological exposure at the local scale is the proper selection of measurement points. It is crucial to obtain a statistically valid sample to avoid overestimation and underestimation errors. The distribution of sampling points based on a uniform spatial distribution does not reflect the actual condition, due to local factors. Among others, the varied geology of the terrain has a significant impact [8,9]. This article will discuss the geological correlations that affect the concentration of radionuclides in soils.

In general, the purpose of this work was to give a geological context to a typical radioecological study of soils, connecting the radioactivity of mentioned radioisotopes with their bedrock matrices. The specific goals of this work were to determine the radioactivity of the natural radionuclides in soil samples, evaluate the absorbed radiation dose, perform radiological risk assessment and find the correlation between variables. The proposed methodology will allow the use of existing geological maps, which could be crucial to forecast the occurrence of natural radioisotopes in soils and to determine new measurement points.

It should be mentioned that there is lack of solutions in forecasting terrestrial radioactivity. If our methodology does not allow us to obtain direct values, the maxima and minima of field radioactivity can be predicted based on it, simplifying further studies.

## 2. Average Radioactivity of Primordial Radioisotopes in Various Rock Formations

Since soil is formed as a result of rock weathering, the radionuclide content of natural origin should be found in relation to the bedrock that forms it. Therefore, the dependencies present in the rocks themselves will be discussed first. The assessment of direct exposure to external radiation is calculated based on an index that takes into account concentrations of ^40^K, ^238^U and ^232^Th, thus, the following discussion will be focused on these components.

Potassium is the seventh highest component (2.83%) by weight content in the Earth’s crust [10]. The high content of potassium and its wide dispersion make the ^40^K radioisotope, the natural abundance of which is 0.0117%, one of the most radioactive of the isotopes mentioned above [11]. Minerals with a high potassium element content include nepheline, potassium feldspars, leucites, glauconites, orthoclase and sylvinite, among others [12]. In rocks, the most significant potassium concentration is observed in igneous rock formations. For example, in granitoids, which have an average potassium content of 3.5%, which corresponds to radioactivity of about 1100 Bq·kg^−1^ (^40^K). Sedimentary rocks generally have lower ^40^K radioactivity, between 63–400 Bq·kg^−1^, depending on the content of minerals of biological origin [13]. A summary of the ^40^K content in rocks is presented in Table 1 along with other radioisotopes.

Uranium radioisotopes (^238^U and ^235^U) are relatively common, but highly dispersed, so they are present in small amounts in rocks, soil and water. The average uranium content in the Earth’s crust is about 2.8 ppm [14]. Due to the abundance of ^238^U at about 99.3%, and the constant ratio to the others, this radioisotope will be considered for the following relations [15]. In rocks, the uranium isotope content is very variable; in those with a higher silicon content (acidic), e.g., granites, concentration is found to be above average, up to 20 ppm. On the contrary, alkaline basalts, for example, contain uranium contents below average. Sedimentary rocks (with the exception of phosphorite) generally contain minimal amounts [13]. Furthermore, uranium is found in the form of high content deposits, such as uranium blend, where the content reaches up to 60% pure uranium [16]. Uranium in the Earth’s top crust can exist in two oxidation stages, U^4+^ and U^6+^ [17]. Oxidized to form the cation U^6+^, it forms soluble complexes with the anions CO_3_^2−^, SO_4_^2−^ and PO_3_^3−^, and in this variant it can undergo leaching and migration. Subsequently, in a rock in which there are reductive conditions, it can form many minerals. The most important minerals in which uranium is the main component are, e.g., uranosphaerite, uranothorit, betaphite and uranothoranite [13]. A summary of the ^238^U content in rocks is presented in Table 1 along with other radioisotopes.

Thorium has seven naturally occurring isotopes, including one primary ^232^Th with an abundance of 98.8% [15]. Like uranium, thorium is widely distributed in the lithosphere, but its average content in the Earth’s crust is 9.6 ppm, more than three times as much [14]. Magmatic rocks have a relatively high thorium content, particularly basalt and dunite, which contain up to 20 ppm [13,18]. Thorium, as a trace component, is found in minerals such as allanite, xenotime, zircon (mineral) and uranium blende. On the other hand, as one of the main components, it occurs in minerals such as monazite, thorite, cheralite and thoranite [13,19]. A summary of the ^232^Th content in rocks is presented in Table 1 along with other radioisotopes.

**Table 1:** Radioactivity of ^238^U,^232^Th and ^40^K in different type of rocks [12,20,21,22,23,24].

Rock	^238^U Bq·kg^−1^	^232^Th Bq·kg^−1^	^40^K Bq·kg^−1^
Kimberlites	39	64	540
Mafic	6	8	210
Gabbro	6–9	8–16	240
Basalt	6–12	6–10	270
Trachybasalt	30	32	810
Andesite	15–30	16–32	540
Dacite	30	40	720
Quartz-porphyry	57	78	1170
Granite	42–60	60–204	1110–1410
Granitoid	21–162	18–52	720–1200
Miaskite nepheline syenite	126	114	1560
Nepheline syenite	174	194	-

## 3. Study Area

Research was carried out for a representative mountain region, which was the Tatra mountain. The Tatras are part of the Central Western Carpathians and are the highest mountain range in the Carpathians and eastern Europe. Due to the geological structure, they are distinguished into The Western and Eastern Tatras, which are additionally divided into the High Tatras and the Belianske Tatras [25]. Due to its unique character and extraordinary landscape values, most of the area of the Polish Tatra Mountains is under protection and is part of the Tatra National Park (TPN). Currently, the TPN covers an area of nearly 220 km^2^ under strict protection, which is the entire area of the Polish Tatra Mountains and the forest complexes located in the park’s buffer zone [26]. The TPN area is located in the Tatra district, with an area of 471.62 km^2^ and a population of more than 68,000 people. The largest concentration of people is the city of Zakopane, located just 2.5 km from the park’s borders and home to nearly 27,000 residents [27]. However, the most important form of anthropopressure on the nature of TPN is tourism. Hiking trails and mountain roads are often visited by tourists. The number of tourists in 2014 exceeded 3 million for the first time and a gradual increase is observed, where in 2019, this number was close to 4 million. Particularly during summer, an increase is evident, when the monthly number of visitors exceeds 0.5 million [28]. However, tourist traffic is limited to trails, and the vast majority of the TPN remains closed.

**Figure 1:**
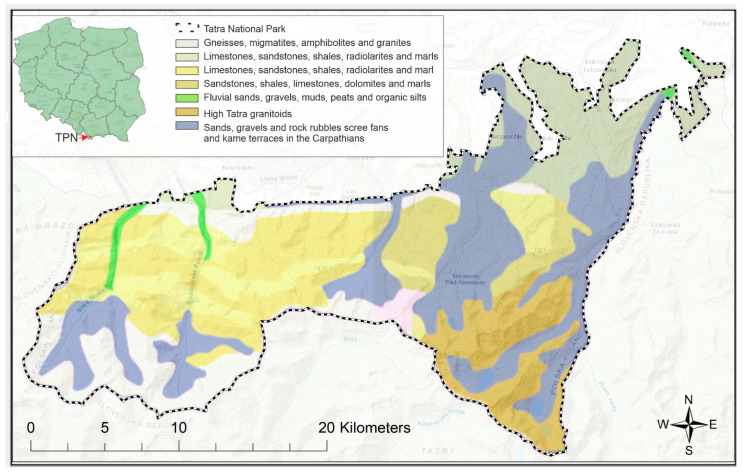
The geological formations in the polish part of the Tatra Mountains based on Polish Geological Institute data [29].

The Tatras are made up of two main forms: crystalline rocks and sedimentary rocks. Crystalline rocks are observed in the High Tatras, while sedimentary rocks are found in the Belianske Tatras. The general distribution of the geological structure of the Tatra Mountains is presented in Figure 1, where a layer of the geological map of Poland at a scale of 1:50,000 is superimposed on the topographic map in ArcGIS software (ArcMAP 10.7.1, ESRI, Redlands, CA, USA) [29]. The Western Tatras are characterized by the presence of both crystalline and sedimentary formations. Among the crystalline formations we distinguish gneisses, granitoids, igneous rocks and metamorphic rocks with granite intrusions. The high resistance to erosion of this type of rock determines the relief of the highest parts, with elevations of 1600–2665 m AMSL [30]. Slow weathering of resistant rocks conditions sharp peaks and ridges. The Western Tatras, on the other hand, are covered by a less resistant variety of crystalline rocks, in the form of metamorphic rocks. The metamorphic rocks of the Western Tatras have a directional structure with layered lining, the course of weathering is easier and involves stratification of the formation. Sedimentary rocks in the Belianske and Western Tatras are mainly limestone and sandstone. In addition, a typical observed rock formation is the overlap of sedimentary rocks on crystalline rocks, which creates the mosaic structure of the Tatras [31].

**Figure 2:**
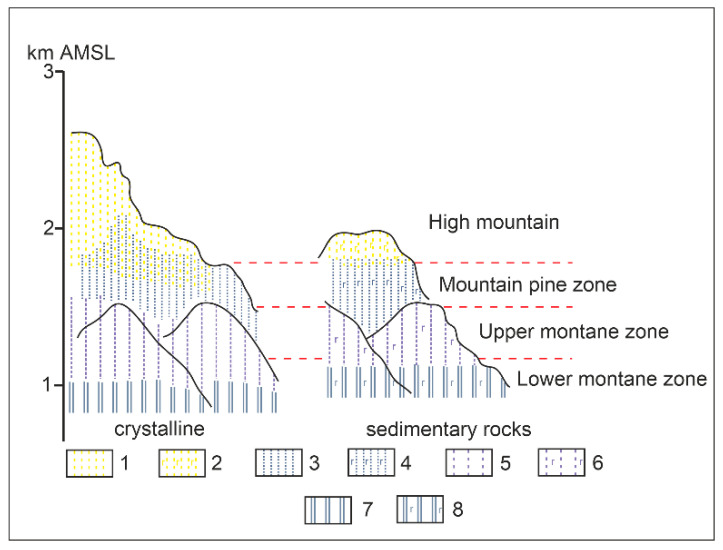
Differentiation of soils in the crystalline and sedimentary rocks of the Tatra Mountains [33,34]. 1—Lithic/Nudilithic Leptosols; 2—Calcaric Lithic/Nudilithic and Hyperskeletic Leptosols; 3—Albic/Protospodic and Dystric Folic Leptosols; 4—Dystric Folic Leptosols; 5—Albic Podzols; 6—Dystric Folic Leptosols; 7—Albic Podzols, Dystric Cambisols (Protospodic); 8—Rendzic Phaeozems, Dolomitic/Calcaric Leptic Cambisols.

Tatra soils are relatively poorly developed, characterized by a high content of skeletic fraction and usually low thickness. Variations are observed depending on the bedrock [32]. Acidic albic podzols and gleyic podzols (humic) were formed on the crystalline core. On the other hand, regarding limestone, the sedimentary rocks are covered by rendzic phaeozems. On sedimentary rocks in the form of sandstone, there are chernozems, cambisols, and leptosols. Soil distribution is strongly influenced by relief, indirectly affecting soil-forming factors as climate, vegetation and water relations. Therefore, the climatic floors outlined are in correlation with the soil floors. Figure 2 shows a diagram of the distribution of soils according to the floor and the characteristics of the bedrock.

## 4. Materials and Methods

### 4.1. Sampling and Preperation

**Figure 3:**
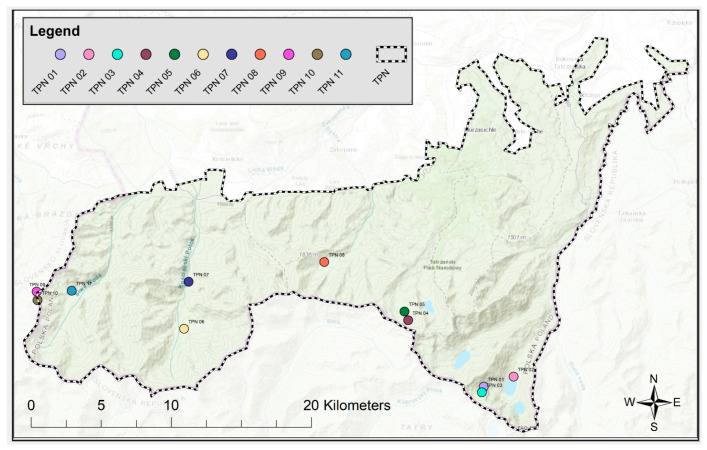
Geographic location of soil sampling points.

**Figure 4:**
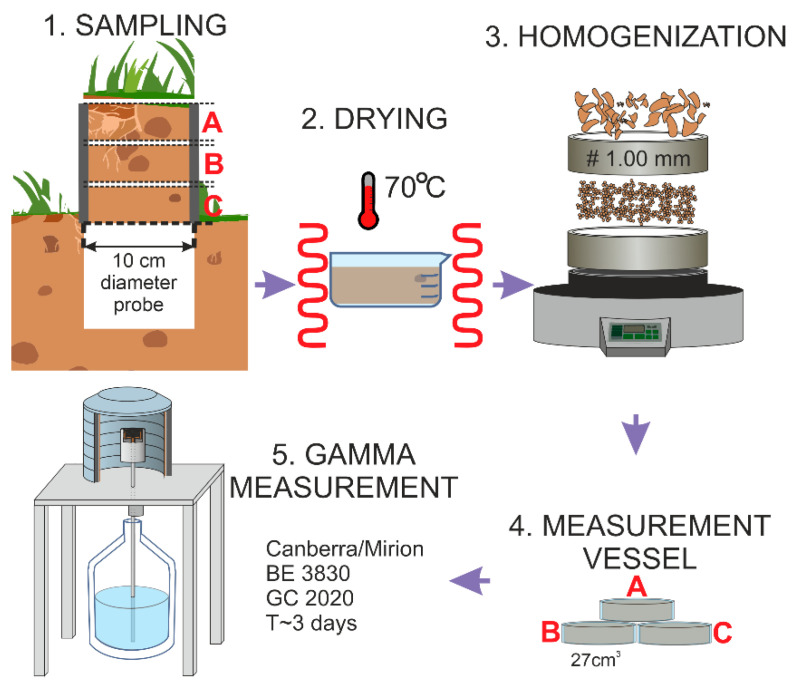
The procedure for sample preparation.

Research dedicated to the distribution of radionuclides in the Tatra Mountains started in 2000. The 11 selected points for the implementation of the assumptions of the presented research have been selected from more than 60 measurement points located in the Tatra Mountains on the Polish side. These 11 points are monitoring points, in which soils are collected every year in order to monitor the radioactivity of the ^137^Cs radionuclide. The locations of these 11 points are shown in Figure 3. From 2015, the procedure for natural radionuclides was developed and implemented. Due to the characteristics of the terrain and often severe weather conditions, it is sometimes difficult to reach the monitoring site and collect the material from all points. In 2017, a complete set of samples was collected, in which the levels of natural radionuclides were also measured, and these points constitute a representative database in this study. Soil samples were collected from the protected area of Tatra National Park. The list of all samples is provided in Table 2. All samples were taken with a special cylindrical probe with an internal diameter of 10 cm and a height of 10 cm, receiving a top layer of soil with a known area. All sampling points were located outside of the routes, in places that are not easily accessible to tourists, avoiding the impact of direct anthropogenic erosion. The consecutively collected material was divided into 3 equal parts, marked as A (depth 0–3), B (depth 3–6) and C (depth 6–10). After transport to the laboratory, the samples were predried at room temperature. The material was placed in a laboratory dryer and dried at 70 °C to dry weight. Subsequently, the soil material was homogenized by mechanical disintegration and sieving through a sieve with a mesh diameter of 1 mm. The measurement geometry was a cylindrical polyethylene vessel with a known volume of 27 cm^3^. The vessels were finally sealed to prevent gaseous decay products from escaping. Soil samples prepared in this way were left for about 30 days to establish a secular equilibrium between ^226^Ra and subsequent isotopes in the uranium-radium series. After this step, the samples were ready for the spectrometric gamma measurement. Figure 4 presents a graphic description of the procedure for preparing soil samples.

### 4.2. Gamma Measurement Details

For measurements, Canberra/MIRION full spectrometric track was used, based on Broad Energy Germanium Detector—BE3830 and GC2020. Performance calibration was carried out by an experiment that determined absolute efficiency (ε) on the basis of three reference materials: IAEA 447 (^40^K); IAEA RGU-1 (Uranium series); IAEA RGTh (Thorium series). In order to determine the radioactivity of individual radioisotopes, the following lines were used:

- 1460.8 keV for ^40^K
- 63.3 keV; 92.3–92.8 keV (^234^Th_equi._) for ^238^U
- 609.3 keV (^214^Bi_equi._) and 295.2 keV; 351.9 keV (^214^Pb_equi._) for ^226^Ra
- 2614.5 keV; 583.1 keV (^208^Tl_equi._), 727.3 keV (^212^Bi_equi._) and 238.6 keV (^212^Pb_equi._) for ^228^Th
- 911.2 keV; 338.3 keV (^228^Ac_equi._) for ^228^Ra

Most of the radioisotopes were assayed assuming secular equilibrium with their progeny. Therefore, the storage period before measurement was set to 30 days. The equilibrium necessary to determine the concentration of ^226^Ra is established during a longest period and is a direct determinant of storage time.

The self-absorption coefficient was measured experimentally following the methodology proposed by Misiak et al. [35]. Measurement uncertainty was calculated based on the following factors:

- uncertainty of peak area calculation;
- uncertainty of the emission probability;
- uncertainty from the sample mass determination.
- uncertainty of self-absorption;
- uncertainty from the background subtraction;
- certified uncertainty from radioactivity of a reference material;
- uncertainty of the efficiency calibration;

Radioactivity mass concentration (*A_M_*) was calculated according to Equation (1), Where *N_S_*—counts number of counts, *N_B_*—subtracted mean background, *m_S_*—mass of the sample, *t*—time of measurement, *p(E)*—probability of emission (*ℇ*)—efficiency, *T_Z_*—self-absorption coefficient.

(1) $$A_{M}\left[Bq\cdot kg^{-1}\right]=\frac{N_{S}-N_{B}}{m_{S}\left[kg\right]\cdot t\left[s\right]\cdot p\left(E\right)\cdot \varepsilon \left(E\right)\cdot T_{Z}}\;$$

The results of the individual depth in the profiles were reduced to the surface emission rate (*Bq∙m^−2^*) to receive a single value *A_S_*. It was calculated by Formula 2.

(2) $$A_{S}\;\left[\mathrm{Bq}\cdot m^{-2}\right]=\frac{A_{M0-3}\left[Bq\cdot kg^{-1}\right]M_{0-3}}{0.0085}+\frac{A_{M3-6}\left[Bq\cdot kg^{-1}\right]M_{3-6}}{0.0085}+\frac{A_{M6-10}\left[Bq\cdot kg^{-1}\right]M_{6-10}}{0.0085}$$

where, *A_S_*—surface emission rate for the depth profile of 0 to 10 cm [*Bq∙m*^−2^], *A*_*M*0–3_, *A*_*M*3–6_, *A*_*M*6–10_—radioactivity in the given layers of soil, *M*_0–3_, *M*_3–6_, *M*_6–10_—total masses of layers, 0.0085 m^2^—surface area of the probe.

### 4.3. Absorbed Dose Rate

To estimate the absorbed dose rate (ADR) in air from terrestrial radionuclides present in soils, conversion factors from *Bq·kg*^−1^ to *nGy*·*h*^−1^ were used. The ADR values take into account exposures by humans to gamma-beta radiation produced by radionuclides in soil which crosses the soil-air interface. Factors were assumed as in USCEAR 2000 report and the ADR calculation method was shown below on Equation (3) [36].

(3) ADR=0.0417 K40[Bq·kg−1]+0.462U238series[Bq·kg−1]+0.604T232hseries[Bq·kg−1]

The value of ^238^*U_series_* in the *ADR* equation was assumed as the highest radioactivity obtained among radioisotopes of a given series. As the value of ^232^*Th_series_*, the mean radioactivity of ^228^Th and ^228^Ra was taken with the assumption of secular equilibrium.

The report proposed taking soil in profiles of 10 cm depth, which did not correlate with the sampling method used. In this case, the method was also applied to track the fallout of anthropogenic isotopes and their infiltration into soil profiles, this was the reason for the sampling differences. Due to the literature gaps, when using the mentioned soil collection methodology, the authors proposed the following solution, which is therefore described in more detail. To integrate fragmentary values with the *ADR* equation, the relevant radioactivity of parts A–C was simplified by the weighted arithmetic mean (WAM). The mean value $\left({\bar{A}}_{0-10}\right)$ takes different contributions of radioactivity of each layer (*A_i_*) by a weight factor equal to total dry mass (*M_i_*) (Equation (4)). It should be emphasized that the weight factor takes a dry mass of the raw layer sample—after drying, before dispersal into measurement vials. The WAM values were calculated separately for each component of the *ADR* equation.

(4) $${\bar{A}}_{0-10}\frac{\sum_{i=0}^{10}M_{i}A_{i}}{\sum_{i=0}^{10}M_{i}}$$

To calculate the uncertainty that includes error propagation of applied ADR formula, the assumption of independent variables was made [37]. Therefore, a formula was simplified and calculated in two steps. The first step of uncertainty was for the WAM (${\bar{u}}_{WAM\;A\;0-10\;cm}$) value and it is shown in Equation (5). The uncertainty of mass determination was recognized as negligible. The solved formula is shown in Equation (6).

(5) $${\bar{u}}_{WAM\;A\;0-10\;cm}=\sqrt{\sum_{i=0}^{10}{\left(\frac{\partial f_{WAM}}{\partial A_{i}}u_{A_{i}}\right)}^{2}}$$

(6) $${\bar{u}}_{WAM\;A\;0-10\;cm}=\;\sqrt{{\left(\frac{m_{0-3}u_{A0-3}}{m_{0-3}+m_{3-6}+m_{6-10}}\right)}^{2}+{\left(\frac{m_{3-6}u_{A\;3-6}}{m_{0-3}+m_{3-6}+m_{6-10}}\right)}^{2}+{\left(\frac{m_{6-10}u_{A\;6-10}}{m_{0-3}+m_{3-6}+m_{6-10}}\right)}^{2}}$$

The uncertainty for the *ADR* formula was solved in the analogous way and is shown in Equation (7).

(7) $${\bar{u}}_{ADR}=\sqrt{{\left(0.0417u_{A\;40K}\right)}^{2}+{\left(0.462u_{A\;238U}\right)}^{2}+{\left(0.604u_{A\;232Th}\right)}^{2}}$$

### 4.4. Geological Map

To correlate the extracted soil profiles with the bedrock, the cartographic study “Detailed geological map of the Tatra Mountains on a scale of 1:10,000” was used. The map is a project of the Polish National Geological Institute, which was developed between 2005 and 2015 [38]. The methodological basis was geological mapping, analysis of previous cartographic materials, as well as laboratory and geophysical investigations (electric resistivity tomography and shallow seismic). Detailed geological mapping was limited to the most problematic regions and those accessible to cartographers. High-resolution numerical terrain models, as well as aerial and satellite imagery, were used for relief analysis [38].

The authors of the article used a shared WMS (Web Map Service) server, and spatial data were processed in ArcGIS, a specialistic software for GIS data analysis. Geoprocessing tools were used for automated spatial analysis of the study areas.

## 5. Results and Discussion

The summary of the measurement results is presented in Table 3. In the table, raw data of the mass radioactivity were presented. The presence of equilibrium in the thorium series was observed. The radioactivity differences of ^228^Ra and ^228^Th were above the value of the measurement uncertainty. Accordingly, the average value of the measurements of both radioisotopes was taken into account in subsequent analyses; the value would be described as *Th_series_*.

**Figure 5:**
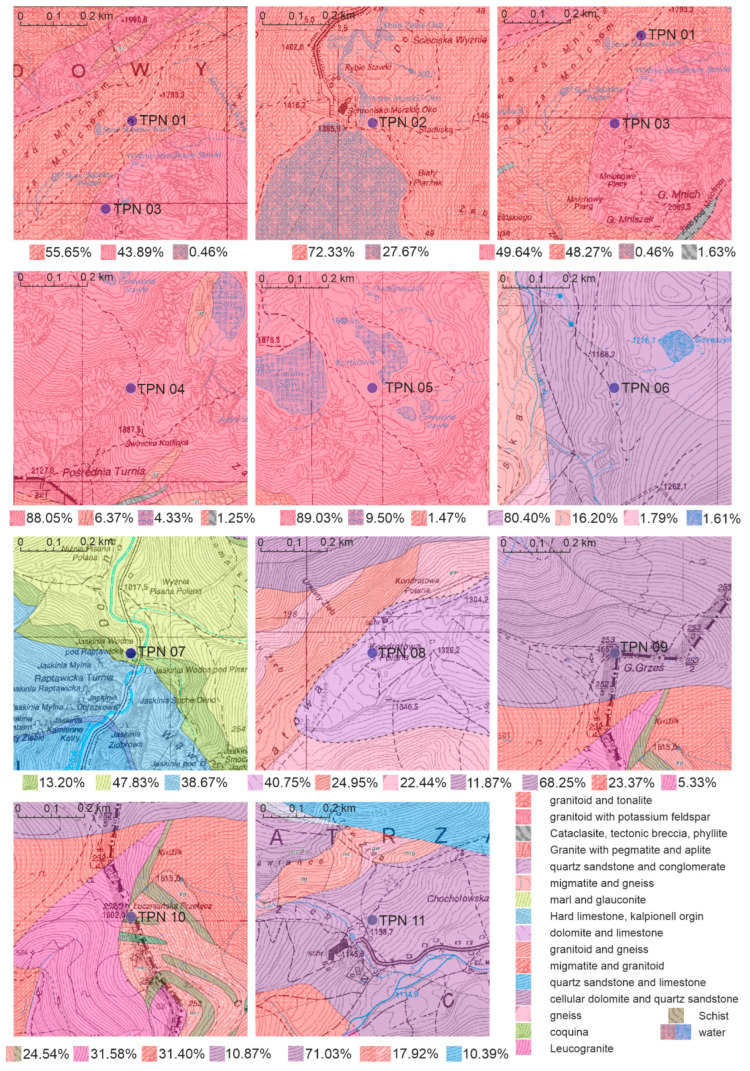
Sampling points with a surrounding area of 0.5 km^2^ and a separate subdivision of bedrock.

Results of the gamma spectroscopy measurements were matched with geological composition data based on GPS coordinates. However, considering that the process of soil formation is not limited to direct contact and is also affected by the surrounding geomorphology, a broader analysis of the bedrock composition was performed, including neighborhood areas. The analysis area was limited to 0.5 km^2^ for each point. The distribution of the bedrock occurrence is presented in Figure 5. The percentage of area covered by the selected rock type was used as a fitting factor between the bedrock in relation to the occurrence of other formations in the assumed fragment of spatial data. In Figure 5, bedrock contribution factors are under each section, and the first value represents a direct component of the bedrock. The most consistent area distributions were at points TPN02, TPN04 and TPN05, with a coefficient greater than 90%, excluding water reservoirs. The bi-componential distribution was observed at points TPN01, TPN03 and TPN06, where the share of other components was negligible. In points TPN01 and TPN03, both components were granitoid type, on the other hand, TPN06 represents mostly quartz sandstone and conglomerate with a 20% share of migmatite and gneiss. Furthermore, a high contribution of the direct component was observed at the TPN09 and TPN11 points, of about 70%. At the remaining points, the distribution was more varied, with the weakest adjustment represented by point TPN07, where the fitting factor was only 13.2%. However, more than 50% of the area at this point consisted of limestone of organic origin.

**Figure 6:**
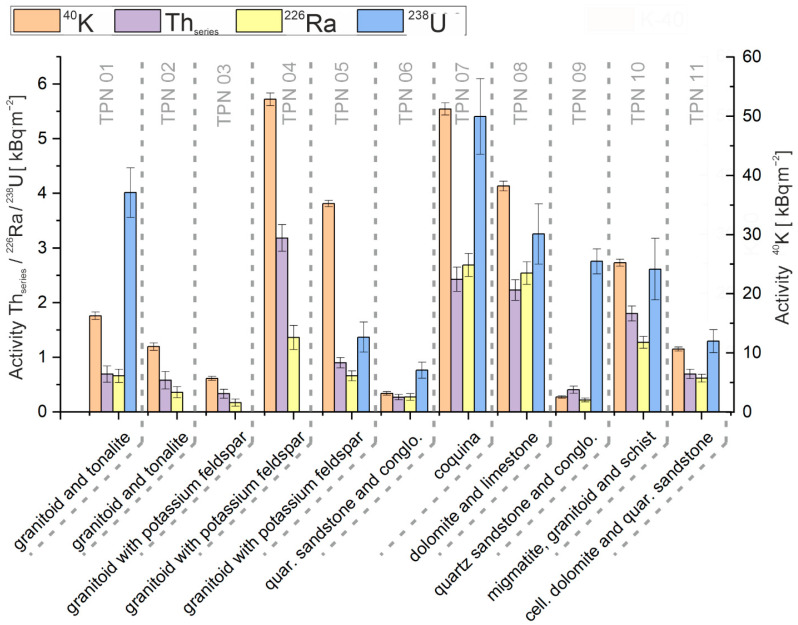
The surface emission rate of individual soil samples with correlation to the bedrock.

Figure 6 shows the radioactivity in units of surface emission rate of individual soil samples with correlation to direct bedrock components. The highest ^40^K radioactivity was observed at the point of granitoids with crystals of pink potassium feldspar (TPN04) and coquina/glauconite (TPN07). The occurrence of potassium feldspars should have a positive contribution to ^40^K radioactivity as a result of the mineralogical composition. The variation may be due to the varying concentration of this fraction in the rock. Special attention should be paid to the point TPN04, which has a large share of the mentioned fraction. At point TPN03, the assigned fraction is identical to point 4, while its share is almost twice as low. When radioactivity is compared, potassium has a value almost ten times higher at TPN04. The reason for this is the likely greater contribution of granitoids to soil formation than would result from a direct relationship to the bedrock. In point TPN07, almost 51.87% of the bedrock was limestone of organic origin, composed of invertebrate species, e.g., shells, mollusks, kalpionell [39]. Consequently, limestones of organic origin should have a higher potassium concentration in the soil. Additionally, the remaining area of TPN07 was covered by the glauconite fraction, which is an iron potassium phyllosilicate. However, the soils formed on limestone fractions generally had a higher concentration of ^40^K. The exception was dolomite, which is a transformed limestone rock, and the values were obtained at an average level of ^40^K surface emission rate. There was considerable variation in ^40^K radioactivity between soils situated in the granitoid layer, with values ranging from 5.61 ± 0.33 kBq·m^−2^ to 35.15 ± 0.52 kBq·m^−2^ (excluding granitoids with pink potassium feldspar crystals). The variation in radioactivity within this group of soils may be due to the content of feldspar. These minerals are the main source of potassium in the soil formed in granitoids [40].

In the case of radioisotopes of the uranium series, ^238^U was the largest radioactivity contributor in most samples. The exceptions were in samples with uranium radioactivity below the MDA level. However, radium was under similar dependence as uranium, with relatively lower radioactivity levels. The highest uranium series isotope radioactivity was observed at point TPN07 of bedrock consisting of organic limestones (coquina, kalpionell), marl, and glauconite with a composition of 51.87% and 47.83%, respectively. Granitoids-soils had varying levels of uranium series radioactivity, ^238^U was in the range of 4.01 ± 0.45−1.37 ± 0.28 kBq·m^−2^ for granitoids. In the granitoid samples below an MDA for uranium, ^226^Ra was in the range of 1.36 ± 0.22−0.17 ± 0.07 kBq·m^−2^. Medium levels of series radioisotopes were observed in samples containing quartz sandstones; in these samples the average uranium radioactivity was 1.60 kBq·m^−2^.

The highest radioactivity of the thorium series isotopes was observed at the TPN04 point, where granitoids with pink potassium feldspar crystals were mainly present. The remaining soils, formed on bedrock composed of granitoids, were characterized by low radioactivity, less than 1 kBq·m^−2^. Soils deposited mainly on organic limestone were characterized by relatively higher *Th_series_* radioactivity.

**Figure 7:**
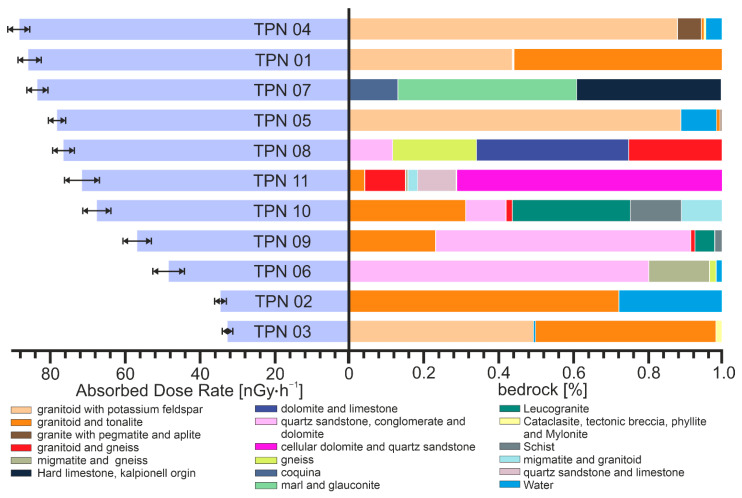
The ADR index for each point, along with a detailed illustration of the bedrock fractions.

Figure 7 shows the ADR index for each point, along with a detailed illustration of the bedrock fractions. Reducing all results to the ADR index allows for observations that are particularly relevant to population radiological protection studies. The points on the chart have been arranged in order of decreasing ADR. The upper part of the graph indicates the higher exposure factors (high ADR), and the significant effect of potassium feldspar granitoids is distinct. However, the fraction had a significant share in TPN03, which is lowest in this graph (the lowest ADR). The probable reason may be the lower proportion of feldspar in granitoids or the specific nature of the soil formation process, for example, the influence of terrain or impact of atmospheric conditions. The fraction of granitoids and tonalites tended to be below the center of the graph. The TPN02 remains the most significant, where the bedrock consists of 100% granitoids and tonalites (after subtracting the water surface). This point has one of the lowest ADRs, which confirms the negative connexon of the fraction concentration with the dose rate. Quartz sandstone is also observed around the intermediate ADR value and is found mainly in the lower value.

In the results obtained, the absorbed dose rate in air of terrestrial origin was on an average level of 62.9 nGyh^−1^ and ranged from 31.0 to 84.5 nGyh^−1^. The mean value for the global distribution is in the range 50–59 nGyh^−1^, depending on the geographic region [41]. For the eastern European region, including Poland, the average value was 59 nGyh^−1^, while for Poland itself the average was 47.4, and was in the range of 18.8 to 86.0 nGyh^−1^ [41,42]. The relative data appear to be consistent with the obtained results.

Exposure to ionizing radiation in relation to natural sources is not obvious, because it is related to the natural characteristics of the terrain. It is hard to relate the ADR rate to just one component—soil. The reference level for public exposure in specific existing exposure situations, e.g., areas of exposure due to residual radioactive material is set as an effective dose—20 mSv per year [43]. The average ADR value obtained for the TPN region (62.9 nGyh^−1^) can be converted to an annual effective dose according to the guidelines of the UNSCEAR 2000 report, which is equal to 0.08 mSv per year [36]. Nonetheless it will take into account only one factor—external exposure in the air due to terrestrial gamma radiation. The reference value takes into account all factors, e.g., internal exposure and intake. However, the values obtained can be compared with the global distribution of ADR for the population. The average ADR value obtained represents the range that 20.2% of the population inhabits, and 5% of the population lives in, an area with a similar ADR as the highest value obtained. Moreover, almost 10% of people live in an area with a higher absorbed dose rate in the air due to terrestrial gamma radiation [41]. Considering these correlations, it can be assumed that the measured activities of natural radionuclides do not differ from the usual ones.

**Figure 8:**
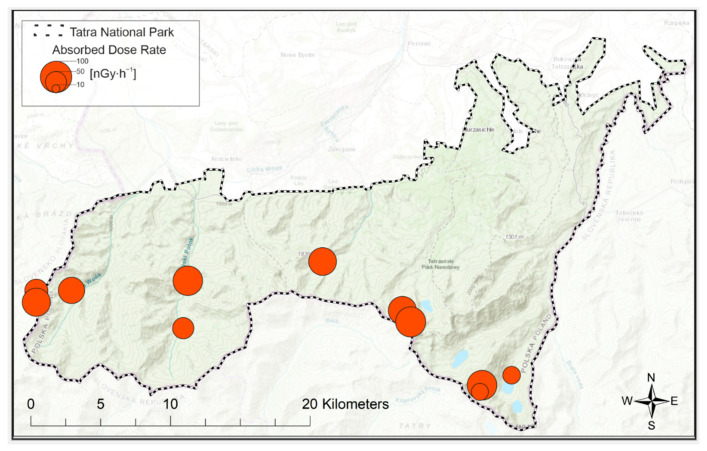
The spatial distribution of ADR in the area of the Tatra National Park.

To show the spatial distribution of ADR, the data is presented in Figure 8. No clear trend is observed in the spatial distribution in the area of the Tatra National Park. ADR averages were 48.7 nGyh^−1^, 71.7 nGyh^−1^ and 62.4 nGyh^−1^, for the eastern, central and western parts, respectively. Therefore, in the eastern part, the average dose is theoretically the lowest, but point TPN02 was the second highest point due to the ADR value. Hence, a spatial analysis does not lead to any conclusions. The probable reason for this is the mosaic geological structure of the Tatra Mountains, the bedrock is not homogeneous, which was already communicated and illustrated in an earlier part of the text. A greater impact on the ADR rate will be related to the contribution of specific bedrock fractions.

In order to find correlations between the composition of bedrock and the soils’ radioactivity, the Principal Component Analysis (PCA) was performed.

**Figure 9:**
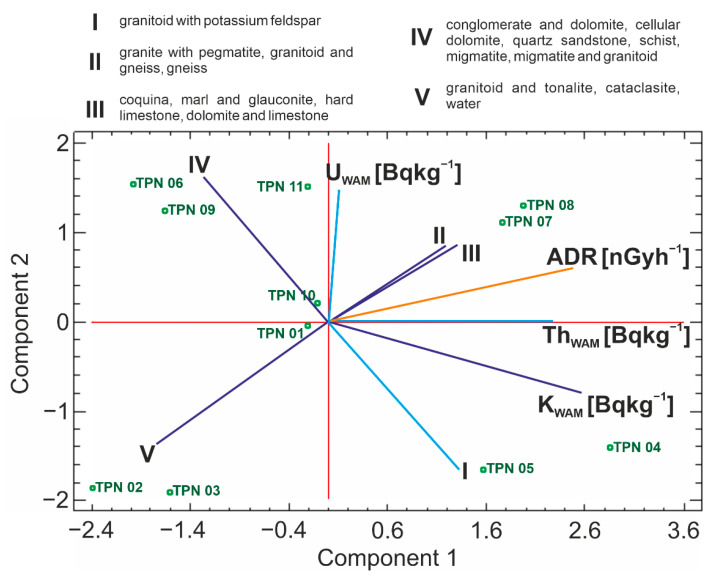
Biplot—Principal Component Analysis.

The PCA was carried out for 11 complete standardized cases using Statgraphics Centurion 18 software, each case represents one measurement point. For the purposes of chemometric analysis, individual components of the bedrock have been grouped, as shown in Figure 8. Groups were created based on similarities derived from the PCA analysis for each of the parameters and gathered to increase the clarity of a biplot (Figure 9). After clarifying the proceedings, the PCA was performed for all components of the bedrock, which showed variables with similar distributions, which were then combined into groups and shown in Figure 9. Within the selected groups, igneous, sedimentary, and metamorphic rocks were observed. Only group number III represented an exclusively sedimentary source.

In Table 4, the eigenvalue, percent of explained variance and cumulative percent of explained variance are presented. Table 5 represents the weights of the components. According to the Kaiser criterion there were four principal components indicated. Taking into account the necessity of data analysis in this work, there is enough to focus on two principal components because they explain almost 60% of the variance.

The biplot with projection of the variables and cases onto the plane of two first components is presented in Figure 9. It can be explained that between the bedrocks’ components from groups I and IV there is the negative correlation. Simultaneously, correlation was found for rocks from group V and group II, as well as group III (e.g., III or/and II decreases and then V increases).

The value of ADR increases with increasing content of the components of groups III and II. It is visible at points TPN07 and TPN08. Based on TPN04 and TPN05, it can be seen that with a simultaneous increase in the fraction of group I and group II, III elements, the increases of K_WAM_ and Th_WAM_ are visible (ADR also increases).

Taking into account the composition of the bedrock, it can be confirmed that the presence of rocks from groups III/II and IV causes an increase of the radioactivity of U_WAM_. This dependence is observable for points TPN11 and also for TPN06 and TPN09, but to a lesser extent. The points TPN06 and TPN09 are also characterized by a low level of K_WAM_.

For points TPN02 and TPN03, the contribution of group V is large and is assigned to a low level of ADR. In this group, water reservoirs were also included, the shares of which proportionally reduced the coefficient of ADR. The relationship seems obvious, however, it shows the impact of reducing a zone of bedrock of which soil is the product of weathering processes. It was an indicator that could verify and confirm that the analysis assumptions were statistically correctly adjusted.

The proposed research methodology allows one to predict dependencies for the study region but is limited to only predicting the maxima and minima occurring in the area. The study points out several types of rock that specifically affect the concentration of radioactive isotopes. However, following the literature values presented in Table 1, a variation is observed for one type of rock. Therefore, depending on the region, the radioactivity of each isotope and the exposure represented by the ADR will vary. Hence, it is impossible to derive a global relationship, and the usefulness of the study is verified only by selecting sampling points. In addition, an obvious limitation is access to geological data for the study site.

## 6. Conclusions

It can be concluded that:

1. geological composition of the bedrock influences the level of the radioactivity in a given area;
2. the groups of specific bedrock components were indicated which contribute the largest share to ADR;
3. the ADR values for the majority of measurements points are a little higher than the mean values of ADR for Poland and the world;
4. in the investigated research area, radiological risk seems to be negligible to tourists, as values do not deviate from globally occurring values and almost 10% of the population lives in areas with higher terrestrial radioactivity. The entire TPN area has not been studied, but based on the sampling points, a significant increase in exposure in the rest of region seems improbable
5. the use of basic chemometric tools on the one hand shows the correlations between variables and on the other hand allows us to make predictions about the range of radioactivity in research area;
6. the proposed method of bedrock analysis appears to be suitable for predicting NORM isotope content. This may help to determine new measurement points and obtain a representative sample during radioecological studies and risk assessment research and;
7. the limitation to 0.5 square kilometers in the analysis of the bedrock, that resulted from the assumption, seems to have been the right approach because it takes into account various forms of terrain.

It is worth emphasizing that this type of research is important, not only for assessing the content of radionuclides in a given area, but also for using these data to estimate the radiological exposure of people present in a given area.

In summary, the proposed methodology is especially applicable to research groups that are not experts in geological science, as it uses only raw data provided by national geological institutions. The study area is used only as an example, but the procedure could be widely applied. The only requirement is to have access to geological data.

## Figures and Tables

**Table 2:** The list of soil samples.

ID	Latitude	Longitude	Altitude [mAMSL]	Label	Depth [cm]
TPN01	49.1969444	20.053333333333335	1789	TPN 01A	0–3
				TPN 01B	3–6
				TPN 01C	6–10
TPN02	49.2011111	20.072499999999998	1449	TPN 02A	0–3
				TPN 02B	3–6
				TPN 02C	6–10
TPN03	49.1945833	20.052194444444446	1853	TPN 03A	0–3
				TPN 03B	3–6
				TPN 03C	6–10
TPN04	49.2246561	20.004876944444444	1849	TPN 04A	0–3
				TPN 04B	3–6
				TPN 04C	6–10
TPN05	49.2283411	20.002531944444446	1689	TPN 05A	0–3
				TPN 05B	3–6
				TPN 05C	6–10
TPN06	49.2210833	19.861194444444447	1242	TPN 06A	0–3
				TPN 06B	3–6
				TPN 06C	6–10
TPN07	49.2408333	19.86416666666667	1054	TPN 07A	0–3
				TPN 07B	3–6
				TPN 07C	6–10
TPN08	49.2490278	19.95111111111111	1348	TPN 08A	0–3
				TPN 08B	3–6
				TPN 08C	6–10
TPN09	49.2366469	19.76671861111111	1630	TPN 09A	0–3
				TPN 09B	3–6
				TPN 09C	6–10
TPN10	49.2330839	19.767203888888886	1606	TPN 10A	0–3
				TPN 10B	3–6
				TPN 10C	6–10
TPN11	49.2370681	19.78908388888889	1213	TPN 11A	0–3
				TPN 11B	3–6
				TPN 11C	6–10

**Table 3:** The results of the gamma spectroscopy measurements.

ID	^40^K [Bq·kg^−1^]	[*u*]	^228^Th [Bq·kg^−1^]	[*u*]	^228^Ra [Bq·kg^−1^]	[*u*]	^226^Ra [Bq·kg^−1^]	[*u*]	^238^U [Bq·kg^−1^]	[*u*]
TPN 01A	251	23	11.6	7.5	13.9	6.6	13.0	3.3	92	20
TPN 01B	478	15	18.2	2.8	18.7	3.4	21.6	3.8	111	11
TPN 01C	535	16	20.4	5.5	22.4	3.5	18.9	3.3	124	11
TPN 02A	297	25	13.2	5.5	18.4	6.7	8.3	2.3	MDA	MDA
TPN 02B	308	20	16.0	6.0	14.8	5.4	12.3	2.6	MDA	MDA
TPN 02C	535	19	25.3	5.1	26.3	4.3	17.0	2.1	MDA	MDA
TPN 03A	203	27	12.6	6.3	15.5	7.9	7.7	2.8	MDA	MDA
TPN 03B	257	18	10.9	4.7	13.0	3.9	8.5	1.9	MDA	MDA
TPN 03C	546	18	31.4	4.7	32.2	4.2	14.3	3.3	MDA	MDA
TPN 04A	204	16	12.9	5.6	10.1	4.9	8.5	3.9	MDA	MDA
TPN 04B	514	18	37.3	5.0	39.5	4.4	16.8	3.4	MDA	MDA
TPN 04C	1204	18	67.4	3.8	68.5	3.6	28.5	3.7	40.6	9.7
TPN 05A	736	17	20.6	6.6	19.0	3.6	16.0	4.4	36	10
TPN 05B	1031	15	22.8	2.9	24.9	2.6	18.6	2.0	38.8	7.6
TPN 05C	1041	15	27.1	4.1	29.2	2.6	20.0	2.9	40.6	7.5
TPN 06A	287	15	9.2	3.5	MDA	MDA	5.9	2.5	MDA	MDA
TPN 06B	196	16	15.4	5.3	14.2	4.6	13.2	4.2	42	13
TPN 06C	97	18	24.1	6.4	25.1	3.9	22.4	3.7	71	11
TPN 07A	607	15	28.2	3.7	30.2	3.3	35.0	3.2	53	14
TPN 07B	710	14	34.9	4.8	32.6	3.1	38.8	2.6	61.5	8.9
TPN 07C	742	14	32.9	4.5	35.3	3.0	37.4	3.0	88.2	8.7
TPN 08A	498	20	23.9	5.9	29.8	5.1	34.2	4.2	37	15
TPN 08B	650	15	38.7	5.1	40.1	3.3	44.9	3.7	59.2	9.6
TPN 08C	729	14	41.4	3.4	41.3	3.1	47.5	3.6	61.1	9.1
TPN 09A	52	12	5.5	2.2	7.2	2.9	MDA	MDA	158	15
TPN 09B	200	15	31.2	5.2	32.9	4.7	18.6	3.6	175	14
TPN 09C	204	14	33.7	4.4	35.0	3.4	21.8	3.2	180	14
TPN 10A	588	15	37.8	4.0	43.2	3.8	29.3	3.0	65	15
TPN 10B	626	14	40.0	3.6	42.5	3.3	30.4	2.6	62	14
TPN 10C	642	14	13.7	3.4	48.4	3.1	35.2	2.4	67	13
TPN 11A	152	22	7.5	3.8	9.8	4.4	7.9	2.3	MDA	MDA
TPN 11B	454	16	27.4	4.3	31.8	4.1	28.5	3.8	55	12
TPN 11C	616	14	36.0	3.3	37.2	3.2	33.3	1.6	82.1	9.1

**Table 4:** Principal Components Analysis.

Component Number	Eigenvalue	Percent of Variance	Cumulative Percentage
1	3.258	36.20	36.20
2	2.090	23.23	59.42
3	1.276	14.18	73.61
4	1.118	12.43	86.03
5	0.643	7.15	93.18
6	0.412	4.58	97.76
7	0.201	2.24	100.00
8	1.05 × 10^−12^	0.000	100.00
9	1.71 × 10^−16^	0.000	100.00

**Table 5:** Table of Component Weights.

	Component	Component	Component	Component
	1	2	3	4
I	0.252	−0.481	0.3687	−0.210
V	−0.334	−0.401	−0.040	0.302
IV	−0.239	0.468	0.0663	−0.519
II	0.228	0.246	−0.514	−0.074
III	0.251	0.248	−0.246	0.645
K_WAM_ [Bq·kg^−1^]	0.489	−0.228	0.0081	−0.010
U_WAM_ [Bq·kg^−1^]	0.022	0.430	0.602	0.257
Th_WAM_ [Bq·kg^−1^]	0.436	0.003	−0.217	−0.325
ADR [nGy·h^−1^]	0.472	0.174	0.352	0.053

## Data Availability

The datasets generated for this study are available on request to the corresponding authors.
